# Quantitative trait loci analysis of seed oil content and composition of wild and cultivated soybean

**DOI:** 10.1186/s12870-019-2199-7

**Published:** 2020-01-31

**Authors:** Yanjie Yao, Qingbo You, Guozhan Duan, Jianjun Ren, Shanshan Chu, Junqing Zhao, Xia Li, Xinan Zhou, Yongqing Jiao

**Affiliations:** 1 Key laboratory of Biology and Genetic Improvement of Oil Crops, Ministry of Agriculture and Rural Affairs, Oil Crops Research Institute of Chinese Academy of Agricultural Sciences, Wuhan, 430062 China; 2 Graduate School of Chinese Academy of Agricultural Sciences, Beijing, 100081 China; 3 State Key Laboratory of Agricultural Microbiology, College of Plant Science and Technology, Huazhong Agricultural University, Wuhan, Hubei 430070 China; 4 Soybean Research Laboratory, Xuchang Research Institute of Agricultural Sciences, Xuchang, 461000 China; 5 Collaborative Innovation Center of Henan Grain Crops /College of Agronomy, Henan Agricultural University, Zhengzhou, 450002 China

**Keywords:** Soybean (*Glycine max*), Oil and fatty acids, QTL, SLAF-seq

## Abstract

**Background:**

Soybean oil is a major source of edible oil, and the domestication of wild soybean has resulted in significant changes in oil content and composition. Extensive efforts have been made to identify genetic loci that are related to soybean oil traits. The objective of this study was to identify quantitative trait loci (QTLs) related to soybean seed oil and compare the fatty acid composition between wild and cultivated soybean.

**Results:**

Using the specific-locus amplified fragment sequencing (SLAF-seq) method, a total of 181 recombinant inbred lines (RILs) derived from a cross between wild soybean ZYD00463 (*Glycine soja*) and cultivated soybean WDD01514 (*Glycine max*) were genotyped. Finally, a high-density genetic linkage map comprising 11,398 single-nucleotide polymorphism (SNP) markers on 20 linkage groups (LGs) was constructed. Twenty-four stable QTLs for seed oil content and composition were identified by model-based composite interval mapping (CIM) across multiple environments. Among these QTLs, 23 overlapped with or were adjacent to previously reported QTLs. One QTL, *qPA10_1* (5.94–9.98 Mb) on Chr. Ten is a novel locus for palmitic acid. In the intervals of stable QTLs, some interesting genes involved in lipid metabolism were detected.

**Conclusions:**

We developed 181 RILs from a cross between wild soybean ZYD00463 and cultivated soybean WDD01514 and constructed a high-density genetic map using the SLAF-seq method. We identified 24 stable QTLs for seed oil content and compositions, which includes *qPA10_1* on Chr. 10, a novel locus for palmitic acid. Some interesting genes in the QTL regions were also detected. Our study will provide useful information for scientists to learn about genetic variations in lipid metabolism between wild and cultivated soybean.

## Background

Soybean (*Glycine max* L. Merr.) is one of the most important protein and oil crops [1], the yields of which accounted for nearly 60% of the world’s oilseed production in 2018 [2]. Soybean oil is mainly composed of five fatty acids, which include palmitic acid (C16:0), stearic acid (C18:0), oleic acid (C18:1), linoleic acid (C18:2), and linolenic acid (C18:3), which are present at approximate average concentrations of 10, 4, 18, 55, and 13%, respectively [3, 4]. The quality of soybean oil depends on the composition of the fatty acids, which affects the nutritional value, flavor, and stability of the soybean oil. Unsaturated fatty acids play an important role in immune system regulation, blood clotting, neurotransmission, cholesterol metabolism, and the structure of membrane phospholipids in the brain and retina [5]. However, unsaturated fatty acids are susceptible to oxidation, resulting in an off-flavor and reducing oil shelf life [6, 7]. Soybeans are thus currently bred to have higher amounts of monounsaturated fatty acids (oleic acid) and lower amounts of polyunsaturated fatty acids (linoleic and linolenic acid), which increases the oxidative stability and is also better for human health [4, 8].

Soybean seed oil content and composition are controlled by multiple quantitative trait loci (QTLs)/genes and are also affected by environmental factors [9, 10]. To date, the QTLs associated with seed oil and fatty acids in soybean have been extensively investigated [11–19]. Since the first research attempted to discover oil QTLs in soybean [10], more than 322 oil QTLs and 228 fatty acid QTLs have been identified across all 20 chromosomes (Chr.) in the SoyBase database [20]. Among these QTLs, some are stable and have been detected in different bi-parental populations and environments, including the QTL regions of 1.64–2.09 Mb and 33.35–35.95 Mb on Chr. Twenty for seed oil content [14, 21–25] and the QTL region of 44.58–48.58 Mb on Chr. Fourteen for seed linolenic acid [11, 13, 24, 26]. However, most of these identified QTLs have low selection accuracy and have not been effectively used in marker-assisted selection (MAS) in soybean for seed oil due to insufficient linkage disequilibrium with desirable QTL alleles and the genetic complexity of the trait [27, 28].

With the completion of the genome sequencing of soybean cv. Williams 82 [29] and the rapid development of next-generation sequencing (NGS) technology, single-nucleotide polymorphism (SNP) markers have been used to construct high-density linkage maps to identify QTL intervals [25, 30]. Based on a high-density genetic map consisting of 2062 SNP markers, Cao et al. (2017) identified one QTL, *qOil-5*, for seed oil, which was mapped to Chr. 05, with a physical distance of 2.5 Mb [30]. Using the Illumina Infinium BeadChip sequencing platform, Patil et al. (2018) reported stable QTLs for oil content on Chr. 02 (*qOil_02*), Chr. 08 (*qOil_08*), Chr. 15 (*qOil_15*), and Chr. 20 (*qOil_20*) using 3343 polymorphic SNPs (3 K-SNP) [25]. Specific-locus amplified fragment sequencing (SLAF-seq) technology has been used to construct high-density genetic maps, constituting an efficient method for large-scale de novo SNP discovery and genotyping in soybean [31, 32]. Li et al. (2017) detected 26 stable QTLs for five fatty acids using 3541 SLAF markers with an average distance of 0.72 cM [31]. Zhang et al. (2018) created a high-density genetic map containing 8597 SNP loci with an average distance of 0.57 cM, from which two QTLs, *qOil10–1* and *qOil10–2*, for oil content were mapped [32]. In addition, some genes associated with soybean seed oil content and composition have been discovered through genome-wide association studies (GWAS) by SNP genotyping [30, 33–38]. All these genes provide useful information for the improvement of seed oil in soybean breeding programs.

In soybean, the key functional genes associated with lipid biosynthesis have been investigated, including the fatty acid desaturase genes *FAD2-1A* (*Glyma.10 g278000*), *FAD2-1B* (*Glyma.20 g111000*), *FAD3A* (*Glyma.14 g194300*), *FAD3B* (*Glyma.02 g227200*), *FAD3C* (*Glyma.18 g062000*), *FAD7* (*Glyma.18 g202600* and *Glyma.07 g151300*) [39–41], 3-ketoacyl-ACP synthase II genes (*KAS II*, *Glyma.17 g047000* and *Glyma.13 g112700*) [42, 43], and the diglyceride acyltransferase gene *DGAT* (*Glyma.17 g053300*) [44]. However, overexpression of a single gene could not significantly increase fatty acid biosynthesis flux [45]. It seems likely that lipid metabolism requires the regulation of multiple related genes. Several important transcription factors have also been found to participate in the regulation of lipid accumulation by directly binding to the promoters of lipid biosynthesis genes. For example, the overexpression of *GmNFYA*, *GmDof4*, *GmDof11*, *GmbZIP123,* and *GmMYB73* significantly increases seed lipid accumulation in transgenic plants [46–49]. Studying these factors can improve our understanding of the mechanisms of lipid metabolism in soybean.

Cultivated soybean seeds have an oil content of approximately 18–22%, whereas wild soybean seeds contain about 8–10% oil [25]. In an attempt to identify genes controlling seed oil content and composition, QTL analyses for oil content between cultivated and wild soybean need to be conducted. In the present study, large-scale SNP markers were developed using SLAF-seq to construct the linkage group and map the QTLs controlling seed oil and composition using a population of 181 recombinant inbred lines (RILs) developed from an interspecific cross between wild soybean ZYD00463 (*Glycine soja*) and the cultivated soybean WDD01514 (*Glycine max*). Our results could inform scientists about the genetic variation in seed oil between wild and cultivated soybean during the domestication process and further improve oil quantity and quality by molecular breeding.

## Results

### Phenotypic variation

The seed oil content and five predominant fatty acid compositions of the parents and progenies were determined from 2015 to 2016 in Wuhan, Hubei Province and Xuchang, Henan Province. Table 1 shows that the average seed oil content of WDD01514 is 23.76%, which is approximately two-fold higher than ZYD00463 (11.89%). In terms of seed oil composition, WDD01514 has higher oleic acid (20.57%) and lower linolenic acid (7.60%) content than ZYD00463, which are 11.39 and 16.60%, respectively. Large variations in oil content and composition were observed among the 181 RILs, ranging from 11.36 to 13.11% for oil content, 17.07 to 22.68% for oleic acid content, and 11.05 to 14.41% for linolenic acid content.

**Table 1:** Summary of statistics on seed oil content and composition in four environments

Traits	Year/Site	P1^a^	P2^b^	Max^c^	Min^d^	Mean^e^	SD^f^	CV^g^(%)	*W*^h^	*P*^i^	K^j^	S^k^	*h*^*2*l^(%)
OC	2015/W	10.75	24.40	23.90	10.51	17.25	2.17	12.58	0.99	0.90	0.29	−0.13	92.0
	2015/X	12.22	23.74	23.63	11.49	16.99	1.93	11.36	0.99	0.24	0.53	−0.15	
	2016/W	13.00	24.10	23.18	11.86	17.88	2.16	12.09	0.99	0.93	0.09	−0.08	
	2016/X	11.60	22.81	21.30	10.23	16.31	2.14	13.11	0.99	0.79	0.04	−0.18	
	Mean	11.89	23.76	23.00	11.02	17.11	2.10	12.29					
PA	2015/W	11.30	10.79	13.43	9.94	11.59	0.61	5.25	0.99	0.45	0.42	0.26	91.2
	2015/X	10.99	10.57	13.32	10.17	11.75	0.63	5.37	0.98	0.17	−0.33	0.26	
	2016/W	11.32	11.34	14.08	10.63	12.03	0.62	5.19	0.98	0.24	0.33	0.42	
	2016/X	11.44	10.90	14.37	10.86	12.49	0.69	5.56	0.98	0.38	0.11	0.29	
	Mean	11.26	10.90	13.80	10.40	11.97	0.64	5.34					
SA	2015/W	3.58	3.58	4.58	2.54	3.63	0.29	8.07	0.99	0.38	0.77	−0.06	80.2
	2015/X	3.37	4.69	4.73	2.96	3.57	0.28	7.81	0.96	0.00	1.41	0.79	
	2016/W	3.55	3.76	4.60	2.91	3.67	0.30	8.15	0.99	0.86	0.23	0.07	
	2016/X	3.49	3.90	4.78	3.21	3.80	0.29	7.51	0.98	0.21	0.24	0.38	
	Mean	3.50	3.98	4.67	2.91	3.67	0.29	7.89					
OA	2015/W	9.95	19.39	35.23	8.16	14.66	3.33	22.68	0.90	0.00	7.72	1.71	86.5
	2015/X	13.11	22.91	32.28	11.24	16.93	3.11	18.35	0.92	0.00	3.93	1.33	
	2016/W	11.17	18.80	24.66	8.56	13.17	2.25	17.07	0.92	0.00	5.30	1.38	
	2016/X	11.33	21.19	31.17	10.44	16.31	2.91	17.83	0.93	0.00	4.10	0.99	
	Mean	11.39	20.57	30.84	9.60	15.27	2.90	18.98					
LA	2015/W	52.40	54.79	59.22	42.63	55.51	2.20	3.96	0.90	0.00	6.13	−1.61	84.0
	2015/X	52.91	52.32	58.83	44.02	54.27	2.17	3.99	0.93	0.00	3.72	−1.19	
	2016/W	52.30	54.72	59.69	47.74	56.09	1.73	3.09	0.94	0.00	3.81	−1.12	
	2016/X	53.06	53.06	57.86	45.14	53.82	2.06	3.83	0.95	0.00	1.17	−0.43	
	Mean	52.67	53.72	58.90	44.88	54.92	2.04	3.72					
LNA	2015/W	18.21	7.71	15.79	5.27	11.08	1.59	14.37	0.98	0.18	0.56	0.12	89.3
	2015/X	15.96	7.11	15.44	5.86	10.66	1.51	14.18	0.99	0.53	0.50	0.12	
	2016/W	15.88	8.02	14.30	8.13	11.06	1.22	11.05	0.99	0.69	−0.20	0.17	
	2016/X	16.36	7.56	12.40	5.77	9.55	1.38	14.41	0.98	0.10	−0.57	0.02	
	Mean	16.60	7.60	14.48	6.26	10.59	1.43	13.50					

^a^The male parent (ZYD00463); ^b^The female parent (WDD01514); ^c^The maximum value of phenotypic data; ^d^The minimum value of phenotypic data; ^e^The average value of phenotypic data; ^f^Standard deviation; ^g^Coefficient of variation; ^h^Shapiro-Wilk (*w*) test for normality; ^i^Significance level of 0.05 for Shapiro-Wilk (*w*) test; ^j^Kurtosis of the of the phenotypic trait; ^k^Skewness of the of the phenotypic trait; ^l^Broad-sense heritability estimated based on the ANOVA in combined environment

The normality test using the Shapiro-Wilk (*w*) statistic indicated that oil content, palmitic acid, stearic acid (except 2015/X), and linolenic acid were normally distributed with *P*-values > 0.05. However, oleic acid and linoleic acid were not normally distributed (*P* < 0.05) (Table 1; Additional file 1). Oleic acid was skewed toward ZYD00463, while linoleic acid was skewed toward WDD01514 (Additional file 1). Moreover, transgressive segregation was observed in the progenies for palmitic, stearic, oleic, and linoleic acid (Table 1; Additional file 1), suggesting that comprehensive genetic recombination of alleles had occurred between parents.

The broad-sense heritability (*h*^*2*^) of the oil and fatty acids ranged from 80.2 to 92.0% in the combined environment, which indicated that the genetic variations accounted for a major proportion of the observed phenotypic variations (Table 1). ANOVA showed that the *F*-value of the G × E interaction was significant for all traits (*P* < 0.001). However, the *F*-value was less than the genotype (Table 2).

**Table 2:** ANOVA of seed oil content and composition over four environments

Traits	Source	Genotype	Environment	Genotype × Environment	Error
OC	DF^a^	180	3	425	1216
	MS^b^	36.99	157.60	2.96	0.11
	*F*^c^	323.29^**^	1377.35^**^	25.85^**^	
PA	DF^a^	180	3	425	1216
	MS^b^	3.37	67.94	0.30	0.01
	*F*^c^	376.85^**^	7599.10^**^	33.05^**^	
SA	DF^a^	180	3	425	1216
	MS^b^	0.57	3.34	0.11	0.00
	*F*^c^	540.31^**^	3167.27^**^	107.19^**^	
OA	DF^a^	180	3	425	1216
	MS^b^	67.94	1169.09	9.18	0.09
	*F*^c^	755.47^**^	12,999.63^**^	102.12^**^	
LA	DF^a^	180	3	425	1216
	MS^b^	31.47	444.20	5.04	0.17
	*F*^c^	185.46^**^	2617.74^**^	29.72^**^	
LNA	DF^a^	180	3	425	1216
	MS^b^	17.06	197.42	1.82	0.03
	*F*^c^	666.06^**^	7707.02^**^	71.03^**^	

^**^P < 0.001; ^a^Degrees of freedom; ^b^Mean square; ^c^F value is for determining significance

A positive correlation was observed between oil content and oleic acid in all four environments (*P* < 0.01), whereas a negative correlation was observed between oil content and palmitic acid, oil content and linolenic acid, palmitic acid and oleic acid, oleic acid and linoleic acid, and oleic acid and linolenic acid in all four environments (*P* < 0.01) (Additional file 2). This suggested that the important genetic factors controlling these traits are tightly linked.

### Construction of a genetic map with SNP markers developed using SLAF-seq

The SLAF-seq method was applied to develop SNP markers between the two parents. Ultimately, a total of 11,398 SNP markers distributed over 20 linkage groups (LGs) were used to construct the genetic linkage map (Additional files 3, Additional files 4). These SNP markers encompassed 2913.78 cM of the soybean genome, with a mean distance of 0.26 cM between markers. The genetic distances of 20 LGs spanned from 126.23 cM (Chr. 11) to 226.60 cM (Chr. 03), with mean marker intervals ranging from 0.15 cM to 0.60 cM. The largest LG (Chr. 01) contained 891 SNP markers, while the smallest one (Chr. 13) had 248 SNP markers (Additional file 5).

### QTLs for oil content

**Fig. 1:**
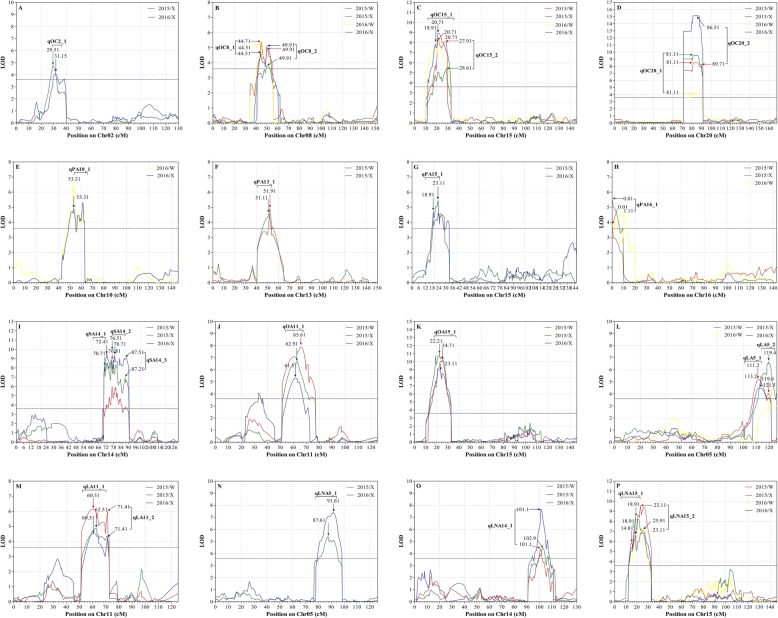
LOD curves on chromosomes for oil content and composition in four different environments. The x-axis indicates genetic position (cM) across the chromosome, the y-axis represents the LOD scores. Horizontal *dashed line* on the chart represents LOD threshold (3.6). Different environments are represented by different lines: *red line* (Wuhan in 2015), *green line* (Xuchang in 2015), *yellow line* (Wuhan in 2016) and *blue line* (Xuchang in 2016). LOD curves for oil content (**a**-**d**), palmitic acid (**e**-**h**), stearic acid (**i**), oleic acid (**j** and **k**), linoleic acid (**l** and **m**), and linolenic acid (**n**-**p**) in different environments. The arrow represents position of a QTL identified under a single environment, and the number is the genetic position (cM) of a QTL on chromosome

**Fig. 2:**
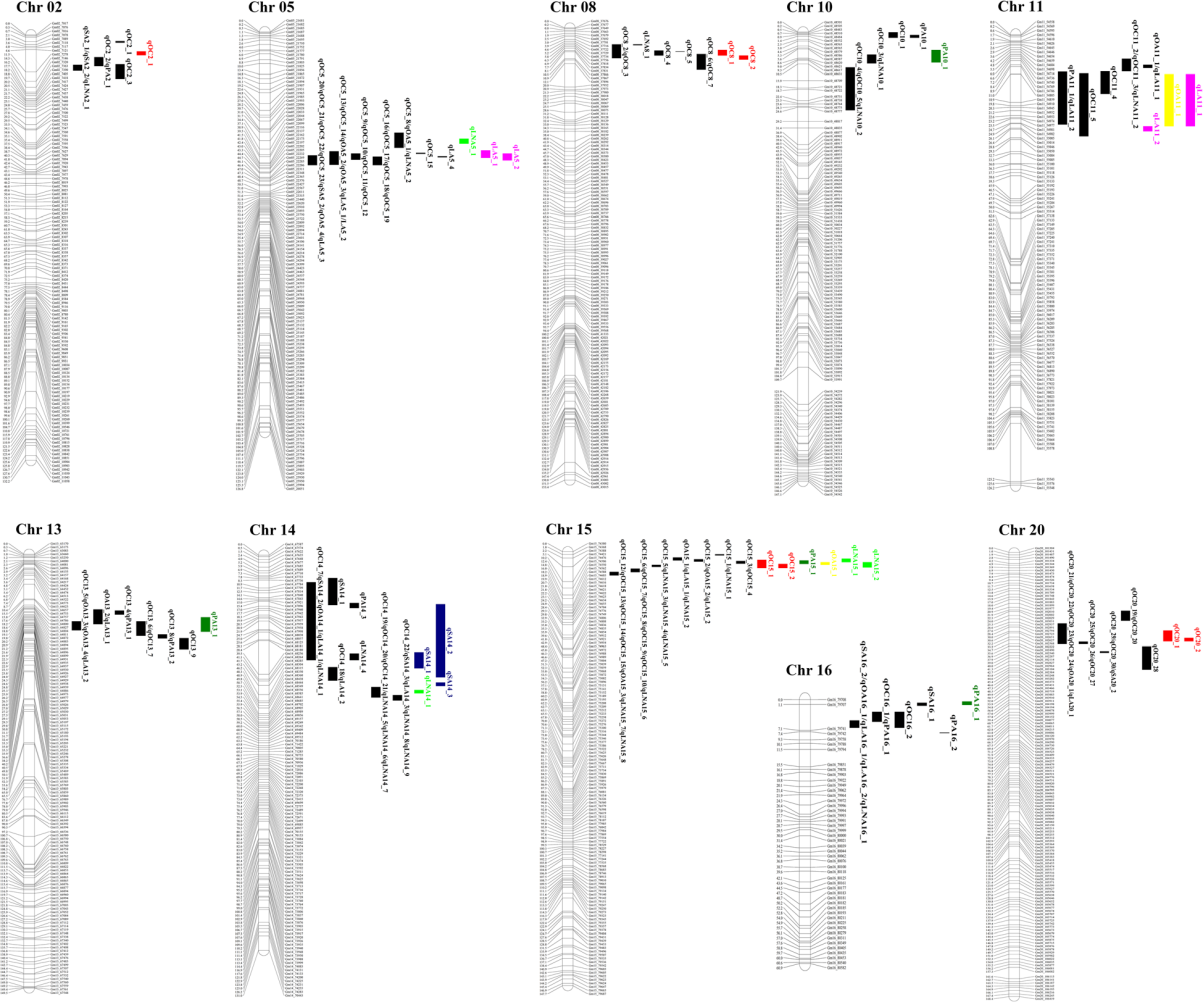
Distribution of stable QTLs in this study and reported QTLs. The QTL regions are marked with bars. The reported QTLs are showed in *black bar*. The stable QTLs in this study are showed in different color bar. *Red bar*: oil content; *green bar*: palmitic acid; *blue bar*: stearic acid; *yellow bar*: oleic acid; *pale red bar*: linoleic acid; *light green bar*: linolenic acid

A total of 22 QTLs for seed oil content were mapped in this study (Additional file 6). Among these, seven stable QTLs with an LOD > 3.6 were identified across multiple environments (Fig. 1; Table 3) and were mapped to Chr. 02 (*qOC2_1*), 08 (*qOC8_1* and *qOC8_2*), 15 (*qOC15_1* and *qOC15_2*), and 20 (*qOC20_1* and *qOC20_2*) (Additional file 7). The QTL *qOC2_1* on Chr. 02 explained an average of 6.7% of the phenotypic variance for oil content; *qOC8_1* and *qOC8_2* on Chr. 08 explained an average of 8.9 and 6.6% of the phenotypic variance, respectively; *qOC15_1* and *qOC15_2* on Chr. 15 explained 12.9 and 11.0% of the phenotypic variance on average, respectively; and *qOC20_1* and *qOC20_2* on Chr. 20 explained 12.2 and 19.3% of the phenotypic variance on average, respectively. All of the QTLs showed negative additive effects, indicating the negative effect on oil content for the allele from the wild soybean parent ZYD00463. In comparison with the reported QTL regions, six QTLs, including *qOC8_1*, *qOC8_2*, *qOC15_1*, *qOC15_2*, *qOC20_1*, and *qOC20_2*, overlapped with previous QTLs (Fig. 2; Additional file 8). The QTL *qOC2_1,* which was located within the interval of 5.08–6.27 Mb on Chr. 02, was adjacent to the mapped QTL within the interval of 6.86–9.67 Mb (Fig. 2; Additional file 8).

**Table 3:** Stable additive QTLs associated with oil content and composition across multiple environments

QTL	Chr^a^	Env^b^	LOD^c^	PVE^d^	Add^e^	Marker Interval	Physical location (Mb)	QTL or QTN^f^
qOC2_1	2	15/16/X	4.1–4.8	6.7	–	Gm02_7476–7548	5.08–6.27	[50]
qOC8_1	8	15/W;16/W/X	4.6–5.4	8.9	–	Gm08_38239–38,423	7.52–9.44	[16, 38, 51, 52]
qOC8_2	8	15/W/X;16/X	3.8–5.1	6.6	–	Gm08_38,423–38,551	9.44–10.8	[51–53]
qOC15_1	15	15/W/X;16/W/X	4.8–8.9	12.9	–	Gm15_74550–74,776	2.80–5.63	[17, 34]
qOC15_2	15	15/W/X	5.4–8.1	11.0	–	Gm15_74,634–74,776	4.15–5.63	[25]
qOC20_1	20	15/W/X;16/W	4.2–9.5	12.2	–	Gm20_104520–105,045	29.7–33.8	[10, 14, 15, 30, 54]
qOC20_2	20	15/W;16/X	8.3–15.1	19.3	–	Gm20_104903–105,045	32.5–33.8	[10, 14, 15, 54]
qPA10_1	10	16/W/X	4.9–6.1	12.9	–	Gm10_49037–49,638	5.94–9.98	novel
qPA13_1	13	15/W/X	4.6–4.9	8.4	–	Gm13_65334–65,803	21.8–26.8	[55]
qPA15_1	15	15/16/X	4.2–5.5	9.7	+	Gm15_74562–74,634	3.00–4.15	[38]
qPA16_1	16	15/W/X;16/W	3.7–5.6	8.3	+	Gm16_79708–79,758	0.42–1.19	[56]
qSA14_1	14	15/16/X	8.2–9.5	18.6	–	Gm14_72100–72,717	32.2–37.5	[33, 37, 56]
qSA14_2	14	15/W/X;16/X	6.0–9.3	16.0	–	Gm14_69722–73,084	16.3–40.5	[36, 38, 56]
qSA14_3	14	15/16/X	6.9–9.1	17.0	–	Gm14_73338–73,548	42.2–43.4	[56]
qOA11_1	11	15/W/X;16/X	5.3–7.9	12.3	–	Gm11_55314–57,241	11.0–25.6	[19]
qOA15_1	15	15/W/X;16/X	9.0–11.0	19.1	–	Gm15_74,608–74,655	3.63–4.45	[57]
qLA5_1	5	15/W;16/X	4.5–5.2	9.9	–	Gm05_25724–25,887	37.5–39.9	[57]
qLA5_2	5	15/W/X;16/W	3.7–6.7	9.6	–	Gm05_25796–25,929	38.5–40.7	[31]
qLA11_1	11	15/W/X;16/X	4.5–6.1	10.4	+	Gm11_55314–57,240	11.0–25.6	[57]
qLA11_2	11	15/W;16/X	4.3–6.0	10.0	+	Gm11_57,240–57,354	25.6–27.0	[57]
qLNA5_1	5	15/16/X	5.4–7.4	12.5	+	Gm05_25415–25,551	33.8–35.4	[18]
qLNA14_1	14	15/W/X;16/X	4.4–7.7	10.0	+	Gm14_73760–73,903	44.7–45.8	[11, 13]
qLNA15_1	15	15/W/X;16/X	5.9–8.5	12.9	+	Gm15_74506–74,608	2.37–3.63	[12, 18, 38, 57]
qLNA15_2	15	15/W;16/W/X	7.0–9.5	16.1	+	Gm15_74,608–74,749	3.63–5.44	[18, 38]

^a^Chromosome; ^b^The four environments were Wuhan, Hubei Province in 2015 and 2016 (15/W and 16/W) and Xuchang, Henan Province in 2015 and 2016 (15/X and 16/X); ^c^Logarithm of odds; ^d^Phenotypic variance explained by the QTLs (%); ^e^Additive effect (%): negative values indicate that the allele from wild soybean parent ZYD00463 has a negative effect on increasing trait values; ^f^The reported QTLs or QTNs for oil content and composition according to the SoyBase database

Six enzyme genes involved in lipid metabolism were identified in these stable QTL regions (Additional file 9), which included two pyruvate kinase genes (*GmPK*, *Glyma.02 g071000* and *Glyma.02 g071100*) within the genomic region of *qOC2_1*, a 3-ketoacyl-ACP reductase gene (*GmFabG*, *Glyma.08 g102100*) within *qOC8_1*, two 3-ketoacyl-CoA synthase genes (*GmKCS*, *Glyma.15 g042500*, and *Glyma.15 g046300*), and a 3-hydroxyacyl-ACP dehydrase gene (*GmFabZ*, *Glyma.15 g052500*) within *qOC15–1*. In addition, two transcription factor genes that might be involved in lipid metabolism were found within the genomic region of *qOC8_2* (Additional file 10), including a homeobox-leucine zipper gene (*GmZIP*, *Glyma.08 g124400*) and a nuclear factor Y subunit A gene (*GmNF-YA*, *Glyma.08 g124200*).

### QTLs for oil composition

A total of 66 QTLs related to seed oil composition were mapped (Additional file 6). Among these QTLs, 17 stable QTLs with an LOD > 3.6 were identified across multiple environments (Fig. 1; Table 3). These QTLs were mapped to Chr. 05, 10, 11, 13, 14, 15, and 16 (Additional file 7). For palmitic acid, four stable QTLs, including *qPA10_1* on Chr. 10, *qPA13_1* on Chr. 13, *qPA15_1* on Chr. 15, and *qPA16_1* on Chr. 16, contributed an average of 12.9, 8.4, 9.7, and 8.3% of the phenotypic variance, respectively. All of the QTLs for palmitic acid, except for *qPA15_1* and *qPA16_1,* showed negative additive effects, indicating the negative effect on palmitic acid for the allele from the wild soybean parent ZYD00463. For stearic acid, three stable QTLs on Chr. 14, namely, *qSA14_1*, *qSA14_2*, and *qSA14_3*, accounted for an average of 18.6, 16.0, and 17.0% of the phenotypic variance, respectively. All of the three QTLs showed negative additive effects. For oleic acid, two stable QTLs, including *qOA11_1* on Chr. 11 and *qOA15_1* on Chr. 15, explained 12.3 and 19.1% of the phenotypic variance, respectively. The additive effect of these two QTLs was negative. For linoleic acid, four stable QTLs, including *qLA5_1* and *qLA5_2* on Chr. 05 and *qLA11_1* and *qLA11_2* on Chr. 11, were identified and explained an average of 9.9, 9.6, 10.4, and 10.0% of the phenotypic variance, respectively. Except for *qLA11_1* and *qLA11_2*, *qLA5_1* and *qLA5_2* showed negative additive effects. For linolenic acid, four stable QTLs, namely *qLNA5_1* on Chr. 05, *qLNA14_1* on Chr. 14, *qLNA15_1* on Chr. 15, and *qLNA15_2* on Chr. 15, explained an average of 12.5, 10.0, 12.9, and 16.1% of the phenotypic variance, respectively. All of the four QTLs had positive additive effects, which indicated the positive effect on these alleles from the wild soybean parent ZYD00463. Among the 17 stable QTLs for fatty acid composition, *qPA10_1* (located within 5.94–9.98 Mb) did not overlap with or was not in the vicinity of the reported QTLs and quantitative trait nucleotides (QTNs) (Fig. 2; Additional files 8 and 11). This suggested that this is a novel locus for palmitic acid.

We identified 21 enzyme-encoding genes that might be involved in lipid metabolism within stable QTL regions (Additional file 9). These genes included two pyruvate kinase genes (*GmPK*, *Glyma.10 g065000* and *Glyma.13 g149800*), two diacylglycerol acyltransferase genes (*GmDGAT*, *Glyma.13 g106100* and *Glyma.13 g118300*), three phospholipid diacylglycerol acyltransferase genes (*GmPDAT*, *Glyma.13 g108100*, *Glyma.16 g005800*, and *Glyma.11 g190400*), two 3-ketoacyl-ACP-synthase I genes (*GmFabB*, *Glyma.13 g128000* and *Glyma.05 g218600*), a 3-ketoacyl-ACP-synthase II gene (*GmFabF*, *Glyma.13 g112700*), two 3-ketoacyl-CoA synthase genes (*GmKCS*, *Glyma.15 g042500* and *Glyma.15 g046300*), a 3-hydroxyacyl-ACP dehydrase gene (*GmFabZ*, *Glyma.15 g052500*), two acyl carrier protein genes (*GmACP*, *Glyma.16 g011300* and *Glyma.05 g201300*), a malonyl-CoA:ACP malonyltransferase gene (*GmFabD*, *Glyma.11 g164500*), an omega-3-fatty acid desaturase 3 gene (*GmFAD3*, *Glyma.11 g174100*), an acetyl-CoA carboxylase gene (*GmACCase*, *Glyma.05 g221100*), an acyl-CoA synthase gene (*GmACS*, *Glyma.11 g194500*), a pyruvate dehydrogenase gene (*GmPDH*, *Glyma.14 g186900*), and a lysophosphatidic acid acyltransferase gene (*GmLPAAT*, *Glyma.15 g034100*). In addition, nine transcription factor genes involved in lipid metabolism were also identified (Additional file 10), including a helix-loop-helix gene (*GmHLH*, *Glyma.05 g200900*), a WRKY protein gene (*GmWRKY*, *Glyma.05 g203900*), a C3H protein gene (*GmC3H*, *Glyma.05 g224400*), two homeobox-leucine zipper genes (*GmZIP*, *Glyma.10 g071700* and *Glyma.11 g145800*), a B3 domain protein gene (*GmB3*, *Glyma.10 g076100*), two MYB protein genes (*GmMYB*, *Glyma.13 g109100* and *Glyma.16 g007200*), and a DBB protein gene (*GmDBB*, *Glyma.15 g029500*).

Co-localization of the QTLs for different traits was also observed on Chr. Eleven and Fifteen (Additional file 7). The QTLs *qOA11_1* and *qLA11_1* were co-located on Chr. Eleven within the physical interval of 11.0–25.6 Mb. The QTLs *qOC15_1*, *qPA15_1*, *qOA15_1*, and *qLNA15_2* were co-located on Chr. Fifteen within the physical interval of 2.80–5.63 Mb.

## Discussion

In the present study, we identified 24 stable QTLs for seed oil and composition. By comparing their mapped regions with previous reports on the soybean reference genome (Fig. 2; Additional files 8 and 11), we discovered that *qPA10_1* did not overlap with or was not adjacent to any of the previously reported QTLs. Furthermore, it did not contain QTNs associated with palmitic acid obtained by GWAS. Due to a higher density genetic map constructed with 11,398 SNP markers, the intervals of the QTL regions were significantly reduced in comparison with those previously reported. For example, for oil content, the physical distance of *qOC8_1*, *qOC8_2*, *qOC15_2*, and *qOC20_2* in our study was 7.52–9.44 Mb, 9.44–10.8 Mb, 4.15–5.63 Mb, and 32.5–33.8 Mb, respectively. In comparison, the intervals were 5.58–10.28 Mb for *qOC8_1* [16], 5.58–10.3 Mb for *qOC8_2* [16], 3.23–4.07 Mb for *qOC15_2* [17], and 27.0–34.3 Mb for *qOC20_2* [54]. For stearic acid, the physical distance of *qSA14_1* and *qSA14_3* in our study was 32.2–37.5 Mb and 42.2–43.4 Mb, respectively. In comparison, the interval was 16.3–45.9 Mb [56]. For linoleic acid, the physical distance of *qLA5_1* and *qLA5_2* in our study was 37.5–39.9 Mb and 38.5–40.7 Mb, while the interval was 37.6–42.2 Mb [57].

In comparison with previously reported QTLs (http://www.soybase.org) (Additional files 8 and 11), 23 out of the 24 QTLs were close to or overlapped with previously reported QTLs (Fig. 2). For example, the QTL *qOC2_1* for oil content on Chr. 02 was located around 5.08–6.27 Mb, which was adjacent to a reported QTL (6.86–9.67 Mb) [50]. In addition, *qOC8_1* on Chr. 08, *qOC8_2* on Chr. 08, *qOC15_1* on Chr. 15, and *qOC15_2* on Chr. 15 were located around 7.52–9.44 Mb, 9.44–10.8 Mb, 2.80–5.63 Mb, and 4.15–5.63 Mb, which overlapped with the reported 5.52–12.64 Mb [16, 51, 52], 5.52–14.21 Mb [51–53], 3.23–4.07 Mb [17], and 4.52–5.21 Mb [25], respectively. For palmitic acid, *qPA13_1* (21.8–26.8 Mb) on Chr. 13 and *qPA16_1* (0.42–1.19 Mb) on Chr. 16 were close to the reported 26.41–29.08 Mb and 2.67–5.06 Mb, respectively [55, 56]. For stearic acid, *qSA14_1*, *qSA14_2*, and *qSA14_3* on Chr. 14 were located around 32.2–37.5 Mb, 16.3–40.5 Mb, and 42.2–43.4 Mb, respectively, which overlapped with the region of 16.30–45.90 Mb [56]. For linolenic acid, *qLNA5_1* (33.8–35.4 Mb) partially overlapped with 31.98–34.65 Mb [18]. Although these QTLs are located in similar regions, whether their responsible genes are identical requires further investigation. In contrast, *qPA10_1* on Chr. 10 was mapped to the region of 5.94–9.98 Mb, which was distal from the reported 0.98–1.87 Mb [24] (Fig. 2; Additional file 8) and did not contain any reported QTNs that were associated with palmitic acid by GWAS [58] (Additional file 11). This result indicated that this QTL is a novel locus for palmitic acid.

By annotating all genes in 24 stable QTL intervals with the Gene Annotation Tool of the SoyBase database [20], 12 important enzyme-encoding genes involved in lipid metabolism were identified (Additional file 9). For example, *GmACCase* (*Glyma.05 g221100*) encoded an acetyl-CoA carboxylase that catalyzes the formation of malonyl-CoA from acetyl-CoA as the direct substrate of the de novo biosynthesis of fatty acids [59]. *GmFabD* (*Glyma.11 g164500*) encoded a malonyl-CoA:ACP malonyltransferase that is responsible for transferring the malonyl group of malonyl-CoA to an acyl carrier protein (ACP). *GmACP* (*Glyma.05 g201300* and *Glyma.16 g011300*) encoded an acyl carrier protein that transports the growing fatty acid chain between the enzymatic domains of fatty acid synthase [60]. *GmFabB* (*Glyma.05 g218600* and *Glyma.13 g128000*) and *GmFabF* (*Glyma.13 g112700*) encoded a ketoacyl-ACP synthases I and II, respectively, which are mainly used to produce palmitoyl-ACP and stearoyl-ACP as the condensing enzyme of fatty acid chain elongation, respectively. *GmFabG* (*Glyma.08 g102100*) and *GmFabZ* (*Glyma.15 g052500*) encoded a 3-ketoacyl-ACP reductase and 3-hydroxyacyl-ACP dehydrase, which catalyze the reduction and dehydration reaction of 3-ketoacyl-ACP, respectively [61]. In addition, *GmFAD3* (*Glyma.11 g174100*) encoded an omega-3 fatty acid desaturase 3 that catalyzes a third double bond into linoleic acid to produce linolenic acid [40, 62]. *GmDGAT* (*Glyma.13 g106100* and *Glyma.13 g118300*) encoded a diacylglycerol acyltransferase that catalyzes the formation of TAGs from fatty acids and glycerol 3-phosphate [63, 64]. The presence of these genes within the stable QTL suggests that these may contribute to soybean seed lipid metabolism. However, whether these enzyme-encoded genes are responsible for the corresponding QTLs requires confirmation using transgenic methods. In addition to the key enzyme genes involved in lipid metabolism, some transcription factor genes also have important roles in regulating fatty acid biosynthesis. We identified 11 transcription factor genes involved in lipid metabolism within the 24 stable QTL intervals (Additional file 10), including *GmB3*, *GmC3H*, *GmDBB*, *GmHLH*, *GmMYB*, *GmNF-YA*, *GmWRKY*, and *GmZIP*. Several transcription factors involved in regulating oil and fatty acid biosynthesis have been studied in soybean. For example, the overexpression of *GmNFYA* in *Arabidopsis* significantly increases seed oil content [46]. *GmMYB73* promotes lipid accumulation in transgenic *Arabidopsis*, possibly through the suppression of GLABRA2, a transcription factor of HD-ZIP [49]. The roles of transcription factors in our QTL regions in the domestication of the seed oil trait in soybean should be further studied.

In the interval of the novel QTL *qPA10_1*, three candidate genes potentially involved in lipid metabolism were also identified, including *GmPK* (*Glyma.10 g065000*), *GmB3* (*Glyma.10 g076100*), and *GmZIP* (*Glyma.10 g071700*). *GmPK* (*Glyma.10 g065000*) encodes a pyruvate kinase (PK). During soybean seed development, phosphoenolpyruvate carboxylase and pyruvate kinase activities contribute to a complex interaction that regulates the metabolic flow of glycolytic carbon into precursors for both protein and oil biosynthesis [65]. In *Arabidopsis thaliana* seeds, reducing the plastidic pyruvate kinase activity by disruption of the gene encoding the β1 subunit of pyruvate kinase resulted in a 60% reduction of seed oil content [66]. *GmB3* (*Glyma.10 g076100*) encodes a B3 domain family of transcription factor. In *Brassica napus*, the *BnFUSCA3* (*BnFUS3*) mutant, a member of B3 domain transcription factors, repressed seed oil levels and increased levels of linoleic acid, possibly due to the reduced expression of *ω-3 FADESATURASE* (*FAD3*) [67]. In soybean, GmLEC2a, a B3 domain transcription factor, complemented the defects of the *Arabidopsis atlec2* mutant in seedling development and triacylglycerol accumulation. The overexpression of *GmLEC2a* in *Arabidopsis* seeds increased triacylglycerol content by 34% and the composition of long chain fatty acids by 4% relative to the control seeds [68]. *GmZIP* (*Glyma.10 g071700*) encodes a homeobox-leucine zipper protein. Song et al. (2013) reported that *GmbZIP123* (*Glyma.06 g010200*) enhances lipid content in transgenic *Arabidopsis* seeds by promoting expression of sucrose transporter genes (*SUC1* and *SUC5*) and cell wall invertase genes (*cwINV1*, *cwINV3*, and *cwINV6*) [48]. In *A. thaliana*, bZIP67 binds to G-boxes in the *FATTY ACID DESATURASE3* (*FAD3*) promoter, enhances *FAD3* expression, and increases linolenic acid seed content [69].

## Conclusions

By means of SLAF-seq technology, we constructed a high-density genetic map comprising 11,398 SNP markers and identified 24 stable QTLs for soybean seed oil content and fatty acid composition using a 181 RIL population derived from a wild soybean and a cultivated soybean. Among these QTLS, one QTL *qPA10_1* did not overlap with or was not close to previously reported QTLs and also did not contain any QTNs associated with palmitic acid, indicating that it is a novel locus. Some interesting genes in the QTL regions were also identified and are worthy of further investigation. Our study provides a valuable information that may contribute to the elucidation of oil biosynthesis in soybean.

## Methods

### Plant materials

The RIL mapping population comprised 181 F_7_ progenies derived from a cross between the wild soybean ZYD00463 (*Glycine soja*) and the cultivated soybean WDD01514 (*Glycine max*). The parents of ZYD00463 (*Glycine soja*) and WDD01514 (*Glycine max*) were provided by the Oil Crops Research Institute of the Chinese Academy of Agricultural Sciences (Wuhan, China). All 181 RILs and their parents were planted at two experimental stations in Hubei Province, Wuhan (N30°35′, E114°33′) and Henan Province, Xuchang (N34°02′, E113°81′) in 2015 and 2016. The two sites possess different climatic conditions, with Wuhan experiencing higher temperatures and more rain and Xuchang experiencing lower temperatures and less rain. Three replicates of the parents and progenies were planted following a randomized complete block design. Each plot comprised a 2.5-m row with 1.0-m spacing between rows and 0.5-m spacing between adjacent plants. For each genotype, seeds were harvested from five plants from each plot at the R8 growth stage (full-maturity stage) [70]. The soybean seeds were air-dried until a constant weight, then the traits were assessed as described below.

### Oil and fatty acid determination

The seed oil content and fatty acid composition were determined according to Wei et al. [71] with minor modifications. Briefly, 30 soybean seeds from each line were ground to a fine powder. Twenty milligrams of each powdered sample were transferred to a 10-mL glass tube. Sulfuric acid-methanol (5%, 2 mL), butylated hydroxytoluene (BHT) (0.2%, 25 μL), methylbenzene (300 μL), and internal standard (IS) (methyl heptadecanoate, Sigma Aldrich, St. Louis, USA, 2.5–5 mg/mL, 100 μL) were added to the samples for fatty acid methyl ester (FAME) preparation, and the sample was esterified in a water bath at 90–95 °C for 1.5 h. After cooling to room temperature, sodium chloride (0.9%, 1 mL) and *n*-hexane (1 mL) were added to the extracts. The supernatant was obtained for gas chromatography (GC) analysis.

The esters were separated by a GC (Agilent 6890 N, USA) fitted with a capillary column (FFAP, 30 m, 0.25 mm i.d., 0.50-μm film thickness). Nitrogen was used as the carrier gas at an inlet pressure of 25 psi. The temperatures of the injection port and detector (FID) were maintained at 250 °C and 260 °C, respectively, and the temperature program for the column was as follows: 210 °C (1 min), increasing to 230 °C at 10 °C/min (22 min). The computer software used for statistical analysis was STATISTICA version 6.0 (Statsoft Inc., Oklahoma, USA). The peaks were identified based on their retention times using authentic standards of fatty acid methyl esters. The relative peak area was used for quantification of the contents of the fatty acids. The formula for calculating seed oil content was as follows:

$$ \mathrm{Oil}\ \mathrm{content}\ \left(\%\right)=\left[\left({\mathrm{A}}_{\mathrm{t}}/{\mathrm{A}}_{\mathrm{s}}\right)\times {\mathrm{m}}_{\mathrm{s}}\right]/{\mathrm{m}}_{\mathrm{i}}, $$

where A_t_ and A_s_ are the total peak area and the internal standard peak area identified based on their retention times, respectively; and m_s_ and m_i_ are the weights of the internal standard and dry seed. The samples of each line were determined thrice.

### Statistical analysis

All the phenotypic data were analyzed using PROC MIXED program in SAS 9.3 (SAS Institute Inc., Cary, NC, USA). Pearson’s correlation coefficients among all traits were calculated from lines in single environments using the PROC CORR function in SAS 9.3.

The heritability in a single environment was estimated as follows:

$$ {h}^2={\updelta}_{\mathrm{g}}^2/\left({\updelta}_{\mathrm{g}}^2+{\updelta}_{\mathrm{e}}^2/\mathrm{r}\right). $$

The heritability across environments was calculated as follows:

$$ {h}^2=\frac{\updelta_{\mathrm{g}}^2}{\updelta_{\mathrm{g}}^2+{\updelta}_{\mathrm{g}\mathrm{y}}^2/\mathrm{n}+{\updelta}_{\mathrm{e}}^2/\mathrm{n}\mathrm{r}}, $$

where ${\updelta}_{\mathrm{g}}^2$ is the genotypic variance component for traits per plot among the RILs, ${\updelta}_{\mathrm{e}}^2$ is the error variance, r is the number of replications for the trait, and n is the number of environments [72, 73]. ANOVA was conducted across environments to determine the significance of genotype, environment, and their interactions. The error components of variance (${\updelta}_{\mathrm{e}}^2$), genotype × environment interaction (${\updelta}_{\mathrm{gy}}^2$), and genotype (${\updelta}_{\mathrm{g}}^2$) were analyzed using the general linear model procedure (PROC GLM) in SAS 9.3 (SAS Institute Inc.). All parameters were estimated from the expected mean squares in the ANOVA.

### Genetic map construction

The SLAF-seq method was applied to develop SNP markers between the two parents [74]. The SNP markers were used to construct the high-density genetic linkage map using the Kosambi mapping function of the JoinMap version 4.0 software [75]. The SNP markers were grouped based on a LOD score of 3.0 and then ordered by the input algorithm to estimate the recombination frequencies. Recombination frequencies between linked loci were transformed into distances (cM) [76]. The collinearity of the LGs with the soybean reference genome was analyzed by aligning the sequence of each SNP marker with the genome sequences of Williams 82 [77].

### QTL mapping and candidate gene prediction

Composite interval mapping (CIM) incorporated into WinQTL cartographer version 2.5 was used to detect additive QTLs [78]. For each trait, the threshold for the identification of a significant QTL with a LOD > 3.6 was estimated by permutation tests with 1000 repetitions at *P* < 0.05. Cofactors were taken into account, and a window size of 10 cM around the test interval was selected for CIM analysis. The distribution of the QTLs on the genetic linkage map was mapped using MapChart version 2.2 [79]. The detected QTLs were denoted by combining a letter or letters representing the abbreviation of traits with a chromosome number [80]. The QTLs that were repeatedly detected in at least two environments, Wuhan and Xuchang across 2 years, were defined as stable QTLs in this study. The QTLs that were previously reported by other groups were defined as reported QTLs. The predicted genes within the stable QTL intervals were obtained from the SoyBase database according to the annotation of the soybean reference genome (Wm82.a2.v1.1). Gene Ontology (GO) enrichment analysis of the predicted genes was performed using the GO website with default settings [81].

## Supplementary information

**Additional file 1. **Frequency distribution of seed oil content and composition in four environments. The *arrows* indicate traits in two parental lines. Wild soybean ZYD00463 is the male parent (P1), cultivated soybean WDD01514 is female parent (P2). The frequency distribution of seed oil content are shown in the chart (A-D), palmitic acid (E-H), stearic acid (I-L), oleic acid (M-P), linoleic acid (Q-T) and linolenic acid (U-X) in four different environments.

**Additional file 2.** Pearson’s correlation coefficients of seed oil content and composition in four environments.

**Additional file 3.** High-density genetic map constructed by SNP markers. The x-axis and y-axis indicate linkage group number and genetic distance (centimorgan, cM), respectively.

**Additional file 4.** Positions of SNP markers on 20 linkage groups and their corresponding physical positions (bp) in the soybean reference genome.

**Additional file 5.** Description of characteristics of the 20 LGs in the high-density genetic map.

**Additional file 6.** Additive QTLs associated with oil content and composition in single environment.

**Additional file 7. **Location of stable additive QTLs on genetic linkage map across environments. QTLs are marked with bars. The bar length represents the physical interval of the QTL. The stable QTLs are showed in different color bar. *Red bar*: oil content; *green bar*: palmitic acid; *blue bar*: stearic acid; *yellow bar*: oleic acid; *pale red bar*: linoleic acid; *light green bar*: linolenic acid.

**Additional file 8.** Information of previously reported QTLs for soybean seed oil content and composition.

**Additional file 9.** Candidate genes involved in lipid metabolism within the 24 stable QTL intervals.

**Additional file 10.** Candidate genes encoding transcription factor involved in lipid metabolism within the 24 stable QTL intervals.

**Additional file 11.** QTNs for soybean seed oil content and composition detected in previous GWAS.

## Data Availability

All data supporting the conclusions of this article are included within the article and its additional files.

## Abbreviations

ANOVA: Analysis of variance
BHT: Butylated hydroxytoluene
CIM: Composite interval mapping
FAME: Fatty acid methyl ester
GC: Gas chromatography
GO: Gene ontology
GWAS: Genome-wide association study
IS: Internal standard
LA: Linoleic acid
LG: Linkage group
LNA: Linolenic acid
LOD: Logarithm of odds
MAS: Marker-assisted selection
NGS: Next-generation sequencing
OA: Oleic acid
OC: Oil content
PA: Palmitic acid
QTL: Quantitative trait locus
QTN: Quantitative trait nucleotide
RIL: Recombinant inbred line
SA: Stearic acid
SLAF-seq: Specific-locus amplified fragment sequencing
SNP: Single-nucleotide polymorphism
