# Supplementary figures and images for: Quantitative trait loci analysis of seed oil content and composition of wild and cultivated soybean
Source: BMC Plant Biol. 2020 Jan 31;20:51. doi: 10.1186/s12870-019-2199-7 (PMC6995124; doi:10.1186/s12870-019-2199-7)

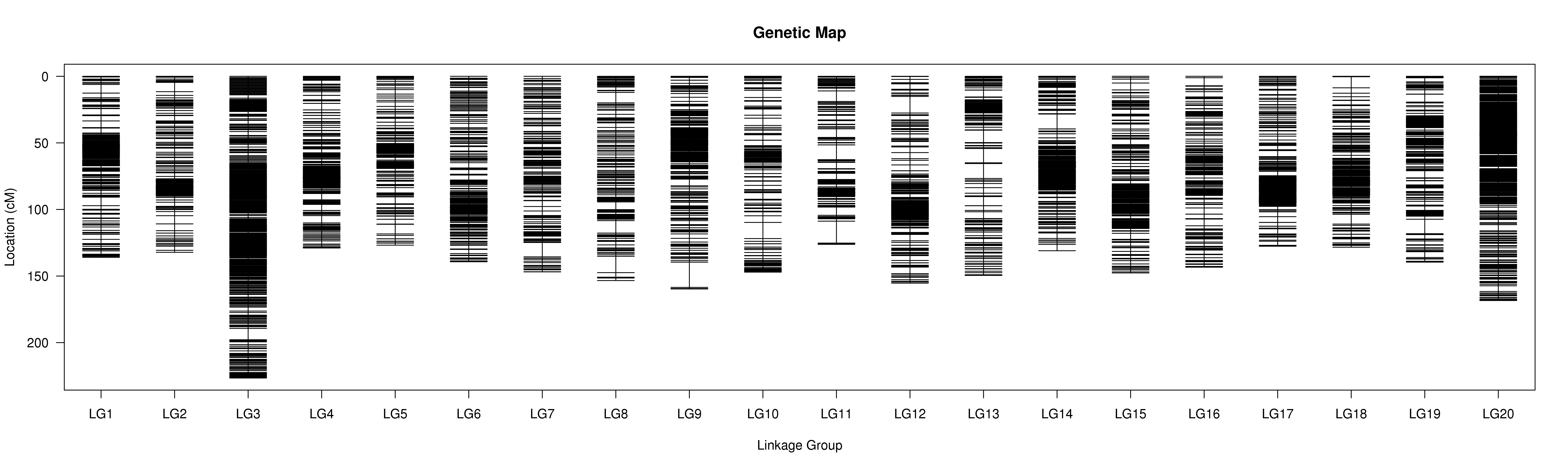

Supplement: Supplementary file 3 — Additional file 3. High-density genetic map constructed by SNP markers. The x-axis and y-axis indicate linkage group number and genetic distance (centimorgan, cM), respectively. [file 12870_2019_2199_MOESM3_ESM.png]

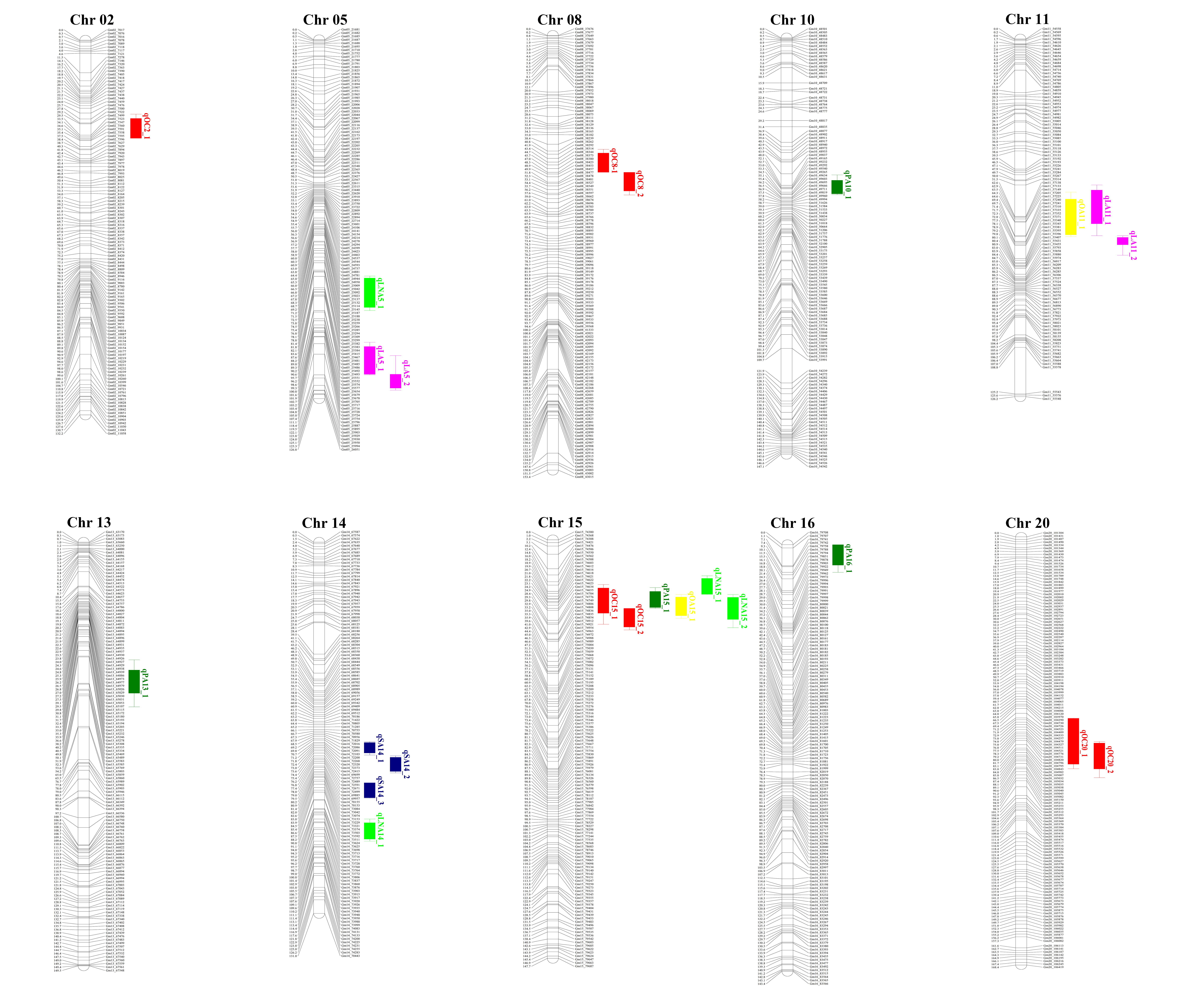

Supplement: Supplementary file 7 — Additional file 7. Location of stable additive QTLs on genetic linkage map across environments. QTLs are marked with bars. The bar length represents the physical interval of the QTL. The stable QTLs are showed in different color bar. Red bar: oil content; green bar: palmitic acid; blue bar: stearic acid; yellow bar: oleic acid; pale red bar: linoleic acid; light green bar: linolenic acid. [file 12870_2019_2199_MOESM7_ESM.png]
